# Advances in current *in vitro* models on neurodegenerative diseases

**DOI:** 10.3389/fbioe.2023.1260397

**Published:** 2023-11-06

**Authors:** Inês Pereira, Maria J. Lopez-Martinez, Josep Samitier

**Affiliations:** ^1^ Nanobioengineering Group, Institute for Bioengineering of Catalonia, Barcelona Institute of Science and Technology, Barcelona, Spain; ^2^ Centro Investigación Biomédica en Red: Bioengineering, Biomaterials and Nanomedicine (CIBER-BBN), Instituto de Salud Carlos III (ISCIII), Madrid, Spain; ^3^ Department of Electronics and Biomedical Engineering, University of Barcelona, Barcelona, Spain

**Keywords:** neurodegenerative diseases, iPSC cell culture, 3D *in vitro* models, bioprinting, microfluidic device

## Abstract

Many neurodegenerative diseases are identified but their causes and cure are far from being well-known. The problem resides in the complexity of the neural tissue and its location which hinders its easy evaluation. Although necessary in the drug discovery process, *in vivo* animal models need to be reduced and show relevant differences with the human tissues that guide scientists to inquire about other possible options which lead to *in vitro* models being explored. From organoids to organ-on-a-chips, 3D models are considered the cutting-edge technology in cell culture. Cell choice is a big parameter to take into consideration when planning an *in vitro* model and cells capable of mimicking both healthy and diseased tissue, such as induced pluripotent stem cells (iPSC), are recognized as good candidates. Hence, we present a critical review of the latest models used to study neurodegenerative disease, how these models have evolved introducing microfluidics platforms, 3D cell cultures, and the use of induced pluripotent cells to better mimic the neural tissue environment in pathological conditions.

## Introduction

Neurodegenerative diseases are defined as the progressive loss of neuronal cells and the synapses formed between them, mainly due to the accumulation of proteins such as amyloid beta, tau, or alpha-synuclein (Kovacs, 2019). One percent of global mortality is ascribed to neurodegenerative diseases. Projections indicate an upward trend in these numbers with each passing year, primarily due to the extended lifespan of the global population. (Badhe et al., 2018). The number of people with dementia in 2018 was 50 million., two-thirds of these patients go for Alzheimer’s while Parkinson’s disease, epilepsy, Huntington’s, and motor neuron diseases like amyotrophic lateral disease (ALS) are the other one-third (Patterson, 2018). As neurodegenerative diseases do not provoke immediate death, patients’ care treatment tends to last a long period, in which their quality of life decreases with a great economic and emotional price. Consequently, patients and their families are under a big financial burden mainly because they not only need medication but also special care provided only by health professionals and external institutions, worldwide estimated cost of 1 trillion US dollars (Patterson, 2018; Cimler et al., 2019). Nonetheless, the numbers for this disease are predicted to triplicate until 2050 and the costs to double by 2030 due to the aging of the population and the increase in the lifespan. (Patterson, 2018).

Even though the discovery of these diseases goes back 100 years, they are still difficult to diagnose and treat (Möller and Graeber, 1998). This is also reflected in the drug development for their treatment. Drug discovery and validation are lengthy and costly processes, mainly due to the strict control necessary to avoid side effects, reducing the number of efficient trials. The billions of dollars invested in drug development are not translated into a high number of drugs approved and the main reason for this is that many preclinical models are not physiologically relevant, failing frequently in the prediction of drug effectiveness in humans (Ahadian et al., 2017). Animal models have been the gold standard in drug development as they allow for testing of drug effectiveness and selectivity in a living organism. However, these models are expensive, time-consuming, ethically questionable, and most of the time unpredictable as clinical trials fail due to the physiological differences between humans and animals (Zhuang et al., 2017). Ineffective drugs must be uncovered in the earlier stages of the process to reduce these costs and the lives of the animals used during the drug development. More efficient drug development can be accomplished through the use of *in vitro* models capable of recreating the physiological characteristics necessary to test the efficacy and safety of a new drug (Breslin and O’Driscoll, 2013). Understanding the architecture of the tissue, the different cells involved and how they interact is key when these *in vitro* models are developed to mimic a biologically healthy/diseased tissue or organ. In this critical review we want to cover the main features involved when developing an *in vitro* model for neurodegenerative diseases, aiming attention to the models that use induced pluripotent stem cells (iPSC) and microfluidic as the most versatile engineering process to mimic human tissue (Garcia-Leon et al., 2019).

## Brain components to be considered within the model

Recreating a reliable *in vitro* model of the brain is a hard task because it is complex to assemble different types of cells, considering cell-cell interactions and the extracellular matrix (ECM). Neurons, despite being the more popular cells, are not the most abundant in the brain, a position occupied by the glia cells family (Barres, 2008). (Fang et al., 2019)This group of cell types encompasses astrocytes and oligodendrocytes, which play roles in supporting and nourishing neurons (Fang et al., 2019). Additionally, microglia protect the neurons and their synapsis, contributing constantly to homeostasis. Increasing evidence suggests that in the case of neuroinflammation, these cells support neuronal destruction and can be active players in the course of neurodegenerative disease (Tejera and Heneka, 2019). Other types of cells found in the brain are the neural stem cells (located mainly in the ventral-hypocavitary zone and the hippocampus) and the endothelial cells. The first can differentiate in any neuronal cell type to replace injured or lost ones and the second ones are the main component of the brain vasculature, being essential for the survival of this organ (Pamies et al., 2014). In the case of the functional units of the Blood-Brain Barrier (BBB), pericytes are also present as they are essential for the maintenance of the barrier functions (van der Helm et al., 2016). The constant communication between these cell types is fundamental for the development and the proper function of the brain but they are not easy to mimic *in vitro*. The ECM is valuable for its specific characteristics, formed by lecticans, proteoglycans with a lectin domain, and a hyaluronic acid-binding domain (Oohashi et al., 2015). The native composition of this matrix is characterized by bioactive cues in components such as laminin that influence neural processes as well as particular mechanical characteristics, such as its stiffness that affect the cells. A particular paragraph about this matrix will follow in the next section.

### Cell types utilized in models of neurodegenerative diseases

Animal cells are the source most used for neurodegenerative disease models and drug development due to their availability and extended knowledge of their phenotypes, but they do not fully predict the complexity of human cells neither in healthy or diseased tissues (Oddo et al., 2019). Primary cells are usually harvested from human or animal tissue (although there are some commercially available options), proving a high functional output and reflecting what happens in physiological conditions (Stacey, 2006). However, these cells are difficult to purify, they grow at a slower rate, and they can lose their phenotype in culture (Potjewyd et al., 2018). As an alternative to them, Immortalized cell lines, defined as primary cells modified to proliferate indefinitely, are widely used for their ability to grow fast, for maintaining their robustness through a high number of passages, and for their low cost in acquisition and maintenance. Even though these types of cells are very useful, the obtained results can be misleading as they do not render a fair replica of all the cells present in the organ, specifically for transporters, tight junctions, and barrier functions which sometimes can present different values depending on the cell line (Stacey, 2006; Stacey, 2006; Stacey, 2006; Rahman et al., 2016; Potjewyd et al., 2018; Potjewyd et al., 2018; Potjewyd et al., 2018)Besides immortalized cell lines and primary cultures, we can find stem cell cultures but their use is often coupled with ethical problems (Bordoni et al., 2018). Adult stem cell extraction is difficult or even not feasible (Zimmermann and Schaffer, 2019).

Then, human induced pluripotent stem cells (iPSC) arise as a promising cell type to develop more reliable neurological *in vitro* models. iPSCs can self-renew and differentiate into any cell type of the three germ layers (Takahashi and Yamanaka, 2006). In this particular case, iPSCs are obtained from somatic cells, healthy humans or patients, that go through a reprogramming process when exposed to a specific cocktail of factors, Oct3/4, SOX2, c-Myc, and Klf4 (Takahashi et al., 2007). This not only allows the construction of a more accurate disease model with cells from diseased patients but, also the evaluation of the response to different treatments for personalized medicine as they contain the genetic information of the patient, which immortalized or primary cells might lack (Poon et al., 2017; Vadodaria et al., 2018).

**FIGURE 1:**
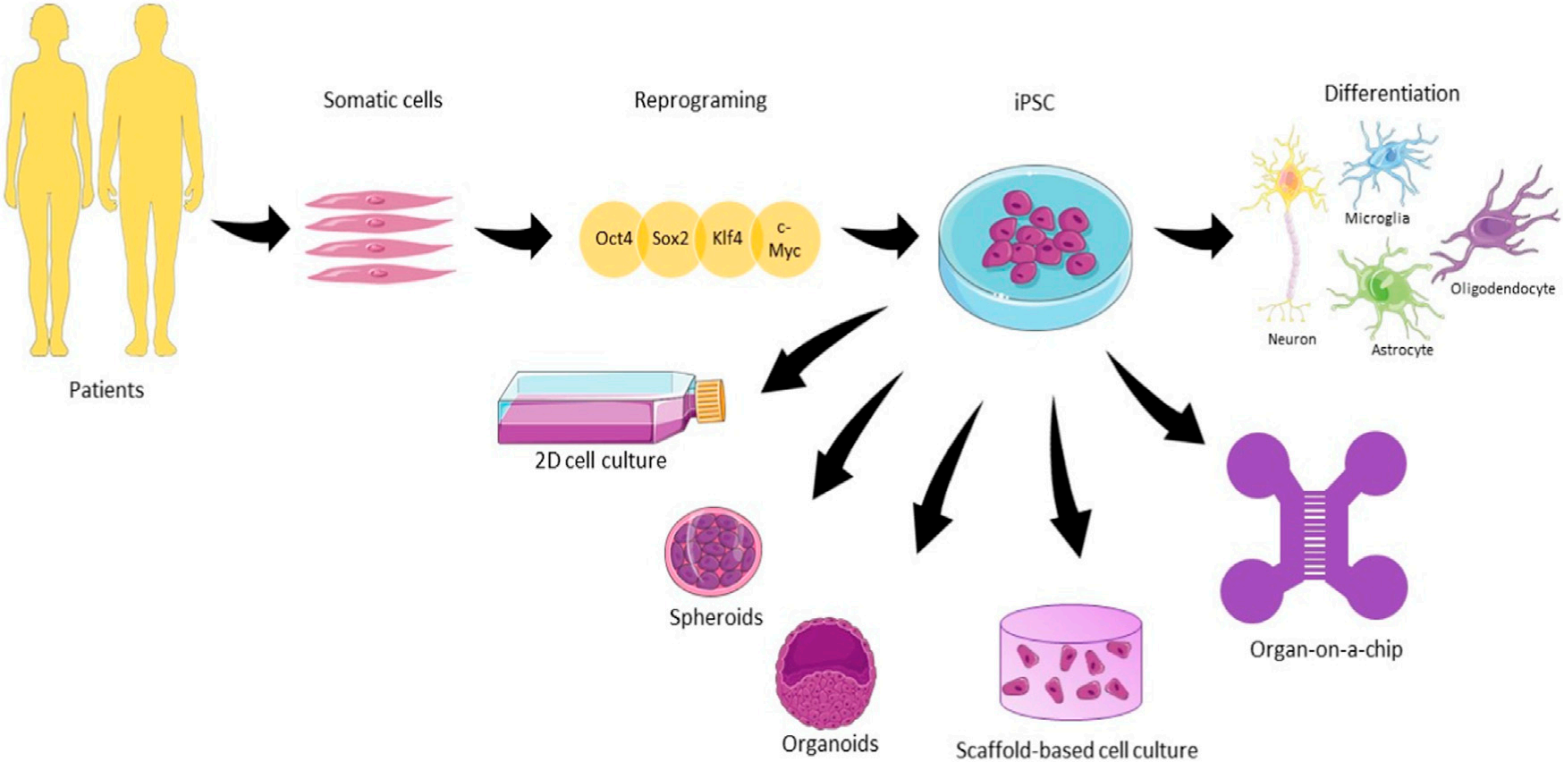
The use of human induced pluripotent stem cells (hiPSC) in different types of neuronal cell culture. Somatic cells are harvested from patients and reprogrammed using the cocktail of factors Oct4, Sox2, Klf4, and c-Myc until the pluripotent state. From that state, iPSC can be differentiated into the main cell types present in the brain. 2D culture plates, spheroids, organoids, scaffold-based culture, and organ-on-a-chip are the main examples of hiPSC culture approached in this review for neurodegenerative diseases (Illustrations obtained from Smart Servier Clinical Art).

Cultivating diverse cell types not only enhances the intricacy of the model but also fosters the proliferation of these cell types, as their behavior is influenced by interactions with other cell types reminiscent of those found in native tissues. (Jeon et al., 2016; Haenseler et al., 2017). There are several available protocols for iPSC differentiation, but they face problems regarding a high variability in the differentiation (Nierode et al., 2019). The enhancement of purity and specificity can be achieved through the application of CRISPR-Cas9 technology, which enables genetic manipulation for the creation or correction of disease-associated mutations, as discussed by Kelava et al. (Kelava and Lancaster, 2016b). However, it is essential to acknowledge that this process is resource-intensive and time-consuming, involving the reversion of cells to a stem-like state and subsequent differentiation into neural cells. Moreover, its complexity and resource demands can further escalate when replicating age-related diseases, impacting the model and potentially leading to hypermethylation (Ghaffari et al., 2018). Figure 1 gives an overview of the iPSCs for different types of models. They differ in terms of (a) their architecture and dimension, (2D or 3D); (b) the absence of scaffolds for spheroids and organoids; (c) the presence of biomaterials representative for scaffold-based cultures; (d) the compartmentalization of the cells culture and (e) presence of physical and chemical cues such as fluid flow offered by microfluidic devices.

### Extracellular matrix

ECM is another key component when building an *in-vitro* model for brain and neurodegenerative diseases because it includes mechanic and chemical cues that influence cell behavior. ECM takes around 20% of the space in the brain. (Rauch, 2004) It is responsible for the low Young’s modulus (around 1 kPa) of this tissue. The glycosaminoglycans (proteoglycans and HA) contribute to the gel-like consistency and viscoelastic properties of this matrix while hydrating it. The low content of elastic fibers and collagen, when compared to other tissues of the body, also contributes to the low stiffness of this tissue (Engler et al., 2006; Shin et al., 2017; Wu et al., 2017)). When building the *in-vitro* model, it is important to replicate the low stiffness of the matrix since neurons tend to prefer softer substrates, but the glia cells prefer stiffer substrates (Her et al., 2013). This divergence is related to the difference of young moduli between the white and grey matter regions of the brain. On the white matter region, the majority of cells are glia and neurons’ axons, being the stiffer region, with an elastic modulus around 1.9 kPa. On the other side, the grey matter region has a lower elastic modulus (around 1.4 kPa) and it is composed of neuronal cell bodies (Budday et al., 2015).

Bioactive cues, such as growth factors or peptides naturally present in the brain ECM, are necessary to mimic the real environment (Gumera et al., 2011). Accordingly, it is common to introduce molecules such as laminin, collagen, or fibronectin in the cell culture as they stimulate cell growth, migration, adhesion, and differentiation. Laminin is one of the most used proteins in brain models since they present specific domains that promote neuronal survival and axonal growth while other domains benefit adhesion and not growth (Edgar et al., 1984; Gumera et al., 2011). Fibronectin and HA are two other examples of bioactive molecules used in neural cultures for their influence on neuron adhesion, migration, guidance, and extension (Tate et al., 2009; Khaing and Seidlits, 2015). The use of small peptides facilitates the integration of the cells while not taking too much space or unnecessary binding sites, as well as avoids disease transmission or batch variation that can appear while isolating the whole protein (Rao and Winter, 2009; Gumera et al., 2011).

In addition to ECM composition, brain tissue architecture, and mechanical factors are also a great influence on neuronal culture. Architecture in a 3D model is documented as extremely valuable for cell-cell interactions along with cell-matrix interactions (Nguyen-Vu et al., 2007; Sun et al., 2007; Kunze et al., 2011; Duval et al., 2017). Related to this architecture, the space and the shape of the compartment where neurons are cultured affect the axon growth since these cells prefer narrow spaces. In addition to topographical cues, the influence of mechanical factors such as stretch and strain plays a significant role in promoting the alignment of neuronal cells and axon growth. The application of fluid flow and shear stress is equally noteworthy in affecting these cultures, especially in modulating their differentiation process. These mechanical factors offer a more realistic simulation of the interactions observed with cerebrospinal fluid in *in vivo* conditions (Pedersen et al., 2007; Rambani et al., 2009; Shin et al., 2009; Peyrin et al., 2011; Choi et al., 2013; Jenkins et al., 2015; Tsao et al., 2015; Mei et al., 2022).

### 3D cell culture models

When cultured in 3D models, cells present biological processes such as differentiation, proliferation, or migration more similarly to what is observed *in vivo* (Baharvand et al., 2004; Liu et al., 2006; Soares et al., 2012). Zhang et al. compared the culture of hIPSC-derived neuroepithelial stem cells culture in a 2D well plate *versus* a 3D self-assembly peptide matrix when studying Alzheimer’s disease and the results prove that the 3D model was more capable of mimicking the pathological side originated by Aβ oligomers (Zhang et al., 2013). We can find different 3D cell culture models in literature for resemble brain and neuropathological diseases (Murphy et al., 2017) and we can classify them according to their fabrication technology, if they use scaffolds as support where cells are seeded on top or embedded in the biomaterial or if the three-dimensionality comes from a cell aggregate (scaffold-free). In the scaffold-based models, the biomaterial is fundamental to introduce mechanical and biochemical cues to the cell culture while in cell aggregates the close interaction of the cells and their self-organization during the differentiation process allows the development of a neural model.

### Scaffold-based cell culture

An ideal scaffold should keep its integrity for enough time to allow cells to self-organize, communicate, and recruit other cells, neurons, or glia. The perfect scenario would be the one presenting a scaffold that stays the time needed for the cells to produce their own ECM to replace it (Knowlton et al., 2018). Several models possess fibers, pores, or channels to stimulate communication within the cells.

The optimal bioaterials for neural culture should be biocompatible, easy to manipulate, capable of chemical or physical modification; allow the permeation of oxygen, nutrients, and electrical conductance; reproducible, and not too expensive (Potter et al., 2008). Taking into consideration the mechanical stiffness and structure of the brain ECM, softer materials are preferred to mimic the consistency of this tissue (Wen et al., 2018). Besides the mechanical strength to support the cells, the scaffold should also have a porosity appropriated for cell migration without compromising its mechanical integrity (Huang et al., 2011).

Hydrogels are one of the most used biomaterials in neural culture. and are described as polymeric networks created through chemical and physical crosslinking with high water content and permeability (Kajtez et al., 2021; Hull et al., 2022). They are biocompatible and flexible in their physical characteristics and composition which allows them to be used in several applications such as cell culture, cell therapy, and drug delivery (Jose et al., 2019; Neves et al., 2020; Poorna et al., 2021). Hydrogels can be tuned to encompass biochemical and biophysical cues present in the brain to promote dynamic interaction between cells and their environment They can be classified as natural, synthetic, or hybrid based on their origin. In Table 1 we show some examples of different types of materials used for the fabrication of scaffolds in neuronal stem cell culture. They are classified by their origin, their production technique, and the cell type used. We also pay attention to their application.

**TABLE 1:** Examples illustrate the use of various polymers in induced pluripotent stem cell models for neuronal culture, each chosen based on its specific application.

Material	Source?	Production technique	Cell type used	Goal	Results	Ref
Collagen type-I	Natural	Unidirectional freezing	Human neuronal progenitor cells-derived astrocytes (hNP-AC), Schwann cells, fibroblast	Evaluation of the influence of 3D collagen scaffold on the growth of hNP-AC axons and cell-cell interactions with Schwann cells, comparing to 2D control	hNP-AC promoted dorsal root ganglion axon regeneration both in 2D and 3D scaffolds. The topography of the 3D scaffold supported the mixing of hNP-AC and migrating Schwann cells from the DRG explant	Führmann et al. (2010)
Hyaluronic acid catechol	Natural	EDC coupling reaction	Human neural stem cells (hNSC)	Development of a HA-catechol biomaterial for hNSC culture	The HA-catechol conjugate showed adhesive and cohesive properties depending on pH. HA-catechol hydrogels are more compatible with neural stem cells compared with other HA hydrogels	Hong et al. (2013)
Matrigel^®^	Natural	-	iPSC-derived neurons with APP or PSEN1 FAD mutations	Creation of an Alzheimer’s Disease 3D model using Matrigel^®^	Aggregation of Aβ was observed after 6 weeks of differentiation. Tau pathology was also observed after 10–14 weeks	Choi et al. (2014)
Alginate and collagen	Natural	Physical mixing and controlled gelation	iPSC-derived cortical neurons	Evaluate the behavior of cortical neurons in a 3D alginate – collagen matrix	The hydrogel’s mechanical properties can be easily tuned. The matrix promoted neuronal differentiation and maturation and the mechanical tuning of the stiffness	Moxon et al. (2019)
Matrigel^®^ and alginate	Natural	Coextrusion microfluidic device with 3D printer	iPSC-derived cortical neurons	Development of a neurodegenerative disease model by encapsulating cortical neurons in matrigel-coated alginate capsules	Cell maturation as well as the switch in Tau splicing during the differentiation procedure was confirmed. A new assay was used to analyze and quantify all the *MAPT* mRNA isoforms	Miguel et al. (2019)
Polyhydroxyphenylvalerate/polycaprolactone	Synthetic	Electrospinning	hiPSC-derived cortical neurons	Evaluate the neurite outgrowth of cortical neurons for the regeneration of the central nervous system using electrospinned nanofibers	The cells’ lifespan increased 2.3-fold, the neurite elongation 3.8-fold and the migration also increased	Cerrone et al. (2020)
Polyethylene glycol diacrylate with Irgacure 2,959 and Dental LT Clear	Synthetic	Two-photon polymerization	iPSC-derived neuronal progenitors	Development of a 3D model for neural network guidance and complex network activity	The scaffold supported the growth, differentiation, and alignment of the cells. The materials could also be patterned and assessed optically	Crowe et al. (2020)
Silk porous scaffolds coated with polyornithine and laminin and filled with collagen	Hybrid	Salt leaching for the porous scaffold, coating, and filling	hiPSC-derived neurons and glia cells	Assemble a 3D model with biological relevance to replicate the 3D neural network formation and function of neurons and glia cells	The model permitted the culture of healthy and patient-derived iPSC and a functional network of interconnected neurons and astrocytes was observed. Stem cells derived from Alzheimer’s and Parkinson’s disease expressed the growth and gene expression similar to the native disease tissue, proving this scaffold to be useful for neurodegenerative disease modeling	Cantley et al. (2018)
Methacrylate-modified hyaluronic acid (HAMA)	Hybrid	Density gradient multilayer polymerization	iPSC-derived neural progenitor cells	Set up a 3D Rett syndrome model and assess the migration and maturation defects of iPSC-derived NPC.	The dysfunction of MeCP2 on the mutant cells used was proven through the differences in migration and maturation of the cells compared with normal iPSC-derived NPC. The 3D scaffold accelerated the maturation and differentiation process of normal iPSC-derived NPC while mature neurons from patients present defective neurite outgrowth as well as synaptogenesis in MeCP2-mutant neurons	Zhang et al. (2016)
Hyaluronan-polyvinyl alcohol (HA-PVA) and alginate-polyvinyl alcohol (AL-PVA)	Hybrid	Inverse emulsion cross-linking technique	iPSC-derived neurons	Compare HA-PVA to AL-PVA biomaterials as scaffolds with tuneable properties for regenerative therapies	HA-PVA and AL-PVA have a similar stiffness to brain tissue. Neuronal growth is enhanced by HA of high molecular weight, low polymer concentration, and brain-mimicking stiffness	Karvinen et al. (2018)
Decellularized porcine brain	Decellularized ECM	Enzymatic digestion for liquid form	iPSC-derived neurons	Use of decellularized matrix for cell culture to evaluate brain injury and neurological disorders	Decellularized brain matrix can be used as a coating for cell culture. iPSC-derived neurons cultured on top of decellularized brains express neuronal markers and present neuronal morphology. This matrix can also be used as a scaffold in gel form	DeQuach et al. (2011)
Adult and fetal decellularized brain	Decellularized ECM	Physical mixing of decellularized ECM with collagen type-I and gelation with NaOH	iPSC-derived neurons and astrocytes	Development of a model for evaluation of iPSC differentiation using decellularized brain ECM.	The differentiation of human neural stem cells to neurons and astrocytes was enhanced when decellularized ECM was used. Specially fetal brain ECM demonstrated to support the long-term maintenance of neuronal differentiation with better results of neuron activity and less toxic reactive astrocytes. The different composition of adult and fetal ECM was tested and native biochemical cues identified	Sood et al. (2019)

Natural polymers are obtained from plants or animals an incorporate the molecules present in the native tissue ECM. Some of the most used natural polymers in neural models are gelatine (Baiguera et al., 2014), alginate (Mosahebi et al., 2001), collagen (Sundararaghavan et al., 2009; Cho et al., 2018), hyaluronic acid (Liang et al., 2013; Wu et al., 2017), Matrigel^®^ (Choi et al., 2014) silk (Hopkins et al., 2013), cellulose (Pértile et al., 2012). However, these materials are difficult to purify and sterilize, processes that can even denature the proteins present in the tissue (Jorfi et al., 2018a). In consequence, there is less control over their physical and chemical properties, as well as their degradation rates, when compared with synthetic biomaterials, presenting differences from batch to batch. Nonetheless, the incorporation of a variety of materials can be advantageous in attaining the intended scaffold structure. (Kuo and Wang, 2013).

Führmann *et al.* used a collagen type-I scaffold for a nerve regeneration model where 2D and 3D architecture were tested for axon growth, thus promoting the effects of human neural progenitor-derived astrocytes found in dorsal root ganglion axon regeneration. The results showed greater axon regeneration on 3D culture when compared to laminin-coated subtract that acted as a positive control. The 3D scaffolds where hNP-astrocytes were seeded with dorsal root ganglion explants showed greater axon regeneration than those without astrocytes. The porosity and the topography of the scaffold also contributed to better communication with the astrocytes, and the migration of Schawn cells and the fibroblast (Führmann et al., 2010). Moxon *et al.* also used collagen in combination with alginate to create a tuneable biomaterial for 3D neuronal culture. The results showed successful incorporation of the collagen fibrils on the scaffold structure as well and adherent human iPSC-derived neurons were capable of creating complex neural networks. The gene expression of the cells was also influenced by the mechanical tuning of the hydrogel stiffness (Moxon et al., 2019).

Inspired by a marine mussel, Hong *et al.* developed a hyaluronic acid catechol biopolymer stable and compatible with neural stem cell culture and with adhesive and cohesive properties depending on the pH. These make this biopolymer interesting for 2D cell culture as well as for 3D when incorporated into gels (Hong et al., 2013). Madl *et al.* produced hydrogels composed of elastin-like proteins with RGD bioactive domains with different stiffnesses and degradability. The influence of these characteristics on neuronal progenitor cells (NPC) stemness was assessed. Remarkably, it was dependent on the degradability and not the stiffness of the substrate (Madl et al., 2017). Later, they also reported NPC cultures in proteolytically degradable hydrogels that express neuronal and astrocytic markers, maturating to neurotransmitter-responsive neurons only when there is degradation of the matrix occurred before the differentiation (Madl et al., 2019).

One of the most natural materials used as a coating in iPSC culture is Matrigel^®^ where human neural progenitor cells have a higher survival rate when differentiating into neurons (Kaiser et al., 2019). Kim *et al.* utilized Matrigel^®^ as a 3D scaffold for the culture of human-derived neurons expressing familial AD mutations. This model could reproduce not only the aggregation of amyloid-β but also the accumulation of hyperphosphorylated tau characteristic of the disease (Choi et al., 2014). Miguel *et al.* also used Matrigel^®^ in the form of capsules coated with alginate for modeling tauopathies. The results identified the switch in Tau splicing along the differentiation of iPSC-derived neurons as well as proved the iPSC-induced neurons were able to differentiate into cortical neurons within the capsules (Miguel et al., 2019).

On the other hand, synthetic materials are more easily tuneable when it comes to their physical and chemical properties as their exact composition is known and can be adjusted. The mechanical properties and degradation rate can be controlled by changing parameters such as molecular weight or crosslinking level. This ability makes them much more reproducible when compared to natural polymers (Zimmermann and Schaffer, 2019). Polyethylene glycol (PEG) (Namba et al., 2009), poly 2-hydroxyethyl methacrylate (Plant et al., 1995), and RADA-16 (Zhang et al., 2013)are some examples. are some examples.

Cerrone *et al.* used electrospinning as a technique to create polyhydroxyphenylvalerate (PHPV) with polycaprolactone (PCL) nanofibers and evaluate their influence on neurite outgrowth of human iPSC. The results showed that this conjugation of synthetic materials increased the cells’ lifespan up to 2.3-fold and the neurite elongation by 3.8-fold. The migration of the cells was also increased when compared to PCL alone (Cerrone et al., 2020). Crowe *et al.* used polyethylene glycol diacrylate with Irgacure 2,959 (PEG2959) and Dental LT Clear (DClear) as suitable biomaterials using a two-photon polymerization technique to create a scaffold for neural culture. This scaffold was biocompatible with the network development of these cells, facilitating observation using imaging techniques. The results showed a scaffold that supported the growth, differentiation, and alignment of the cells while being capable of assessing the individual cells optically (Crowe et al., 2020). Ranjan *et al.* developed a 3D microfibrous scaffold where AD patient iPSC derived NPC was encapsulated in poly (lactic-co-glycolic acid) (PLGA). Results showed reduced cell proliferation and significant acceleration in neuronal differentiation when compared to 2D cultures. In addition, the 3D culture demonstrated higher levels of pathogenic amyloid-beta 42 and phospho-tau in cultures where neurons carried familial AD mutations when compared to heathy neurons (Ranjan et al., 2020).

However, synthetic materials do not possess biomolecules such as adhesion ligands or proliferation and differentiation promotors, limiting the interaction between neural cells and scaffolds. An easy solution for this downside is to undergo biochemical modifications in the materials or combine them with natural polymers to insert biomolecules such as growth factors or adhesion molecules to create a scaffold more similar to the ECM (Aurand et al., 2014). Cunha *et al.* combined RADA 16-I based self-assembly peptides with RGD and laminin-derived motifs, BMHP1 and BMHP2, to create a 3D scaffold for the culture of neuro stem cells. The cells were found to be viable, and proliferative and they differentiated on this scaffold (Cunha et al., 2011). However we need to be aware that the products of synthetic material degradation can become toxic for the cell culture as they do not have a natural origin (Zimmermann and Schaffer, 2019).

The methacrylation of natural polymers, such as gelatin, serves as a facilitating mechanism for the covalent linkage of multiple polymers, whether they are of natural or synthetic origin. (Yue et al., 2015). Hybrid scaffolds combine the best properties of natural and synthetic polymers, being reproducible, easy manipulation of their physical and chemical properties, and high affinity for the cells.

Cantley *et al.* showed the combination of different materials to create a 3D neural model. The scaffold was composed of a sponge of fibrin coated with polyornithine and laminin, natural polymers known to influence neural cell culture, to which collagen was added to support the cell culture. This model permitted the culture of healthy and patient-derived iPSC providing a functional network of interconnected neurons and astrocytes after 5 days of culture. Stem cells derived from Alzheimer’s and Parkinson’s disease patients expressed growth and gene expression similar to the native disease tissue, proving that this scaffold was suitable for neurodegenerative disease modeling (Cantley et al., 2018). Zhang *et al.* developed a model using methacrylate-modified hyaluronic acid to compare the maturation and differentiation of human iPSC-derived neural progenitors from both healthy donors and Rett syndrome patients. The results demonstrated accelerated maturation and differentiation of the healthy cells while MeCP2-mutant cells were defective in migration, having a slower differentiation rate with defective neurite outgrowth and synaptogenesis (Zhang et al., 2016). Karvinen *et al.* created a scaffold adequate for neural cell culture composed of hyaluronan-polyvinyl alcohol (HA-PVA) and alginate-polyvinyl alcohol (AL-PVA). The results proved that the scaffolds were tunable, with a similar stiffness to brain tissue. The presence of HA enhanced the neuronal growth of iPSC-derived neurons (Karvinen et al., 2018).

Another option for scaffold fabrication in cell culture is decellularized ECM. This type of matrix is characterized by the maintenance of the intact native ECM molecules of the tissue without the cellular components where other cell types, including from other species, can be cultured. Removing the original cells present in the tissue allows the escape from the immune and inflammatory responses a scaffold could have when new cells are added to the system while mimicking better the cell environment by maintaining the architecture and biomolecules present in the ECM (Kabirian and Mozafari, 2019). Decellularized ECM can be used the moment after extracting the cells present or lyophilized and pulverized during the purification process and later solubilized to be used as scaffold material (Jang et al., 2017; Santschi et al., 2019). Sood *et al.* studied the difference between using fetal and adult decellularized brains on the differentiation and functional maturation of hiPSC. Fetal ECM brain showed the best results for the maintenance of differentiated neurons with better activity. in long-term culture Astrocytes were also present during the second month of differentiation, proving to be less toxic for the culture. The biochemical cues of both fetal and adult decellularized brains were assessed, showing properties similar to native tissue (Sood et al., 2019).

Obtaining intact decellularized ECM scaffolds can be challenging due to the potential damage inflicted on native components or the alteration of mechanical properties during the decellularization process. DeQuach et al. developed a process to decellularize the porcine brain using detergents capable of keeping several isoforms of collagen, glycosaminoglycans, perlecan, and laminin and be used later in a neural cell culture. This material was proven to be appropriate as a coating of iPSC-derived neurons which expressed normal neuronal markers as well as morphology (DeQuach et al., 2011; Sood et al., 2019) Decellularized ECM can also be coupled with other materials to enhance their performance. Barroca et al. combined decellularized ECM from the adipose tissue with reduced graphene oxide through thermally induced phase separation aided by carbodiimide crosslinking to study the effect of on neural culture since this particular source of ECM had not been exploited for this cell culture. The scaffold proved to benefit from the protein-rich ECM and structural support provided by the reduced graphene oxide where neural stem cells adhered and grown. The concentration of reduced graphene oxide had a direct impact on the cell fate of these stem cells, with higher concentrations inducing neuron differentiation (Barroca et al., 2023).

#### Scaffold-free models

On the other hand, scaffold-free models use cell aggregates, tissue strands, or cell sheets as building blocks for seeding the cells in 3D environment. As this model does not use any material to build its architecture, the organogenesis/embryogenesis process can produce heterogeneous tissues including complicated architectural structures (Jeong et al., 2020).

Spheroids for neuronal models are characterized by an agglomeration of cells, formed spontaneously or by force when they are cultured in a way that they are unable to attach to a substrate material, like the process that naturally happens in embryonic development where cells self-assembled to form more complex tissues (Laschke and Menger, 2017). One of the most interesting features of spheroids is that they together can produce their own ECM as the cells are forced to interact with each other (Dingle et al., 2015). Besides producing ECM, spheroids recapitulate cell-cell interactions and features of natural tissue such as mechanical stiffness and electrophysiology (Laschke and Menger, 2017). These characteristics make spheroids good candidates to model the brain, for performing drug screening, or for modeling a disease. Sloan *et al.* generated and combined neural spheroids to form forebrain assembloids to evaluate the interactions of glutamatergic and GABAergic neurons *in vivo* (Sloan et al., 2018). However, Leite *et al.* used brain spheroids to assess the neurotoxicity of nanoparticles (Leite et al., 2019). Regarding studies of neurodegenerative diseases, Lee *et al.* generated 3D neurospheroids from iPSC of Alzheimer patients to characterize the Aβ generation and drug inhibition (Lee et al., 2016).

The cell organization in spheroids promotes the formation of cell-cell junctions which are crucial to keep the structural integrity of the spheroid while facilitating barrier functions. However, these junctions differ from synaptic junctions which are essential for neural communication and signaling. It is then important to assess the formation of tight junctions and synaptic activity for a complete characterization of the brain model (Wilson et al., 2020; Jurek et al., 2022).

Spheroids can also be used as building blocks, coupling them with scaffolds such as hydrogels, matrigel, or collagen in models for brain disease (Tang-Schomer et al., 2018). Xiao *et al.* used a modified alginate hydrogel to support the culture and differentiation of spheroids generated from neural pluripotent cells derived from mouse iPSC (Xiao et al., 2019). Jorfi *et al.* used iPSC neurospheroids derived from a familiar mutation of Alzheimer’s disease. Spheroids were cultured in Matrigel for 8 weeks and results showed an accumulation of amyloid-β and phosphorylated tau (Jorfi et al., 2018b).

Similar to them, organoids are also formed of cell aggregates, but the cells are capable of self-organizing and differentiating within the aggregate, creating an organ-like structure and mimicking some of its functions. These body structures are more stable, and they can survive for longer periods when compared to spheroids (Brawner et al., 2017). There are two different ways to produce a brain organoid, the undirected method where the stem cells self-organize, or the directed method where the differentiation is externally conditioned by the addition of molecules and factors. The first method originates greater cell diversity within the aggregate while the last allows the regional development of specific organoids (Hartlaub et al., 2019). Then these brain organoids can be fused to study their interactions.

These organoids are used in several types of studies such as fetal development, cell-cell interactions, and/or drug screening (Lancaster et al., 2013; 2017; Quadrato et al., 2017; Zhou et al., 2017; Arlotta, 2018). Bagley *et al.* developed ventral and dorsal forebrain cerebral organoids from hPSCs and co-cultured them to recreate the dorsal-ventral axis. Results showed the migration of GABAergic neurons from the ventral to the dorsal forebrain by using a labeling technique adequate to study neuronal migration (Bagley et al., 2017). Raja *et al.* developed a protocol for the culture of organoids derived from AD patients’ iPSCs which recapitulated characteristic features of the pathology, i.e., hyperphosphorylated tau protein, amyloid aggregation, and endosome abnormalities (Raja et al., 2016). Alternatively, Abud *et al.* used organoids to evaluate the function of microglia-like cells derived from iPSC in neurological diseases; specifically, targeting the effect of Aβ fibrils, tau oligomers, and gene expression, also related to AD (Abud et al., 2017). Wulansari *et al.* developed midbrain-like organoids using hESC with DNAJC6 mutation (identified in early-onset PD patients). The model was capable of replicating midbrain-type dopamine neuron degeneration, aggregation of pathologic α-synuclein, mitochondrial and lysosomal dysfunctions, and increase of intrinsic neuronal firing frequency (Wulansari et al., 2021).

One of the disadvantages of organoids and spheroids is that the center of the organoid can be absent of oxygen and nutrient transportation as the vasculature-like networks are not properly developed, limiting their culture time (Kelava and Lancaster, 2016a; Eglen and Reisine, 2019). This inability to culture organoids for long periods is also a problem for neurodegenerative disease studies. These diseases are characterized by late onset, so the culture time of these organoids might not be sufficient to grow mature neurons and glia cells to display the characteristics of diseased cells (Gopalakrishnan, 2019). To overcome this difficulty, there is a need to find a manner to perfuse the cells with a culture medium. Currently, bioreactors with agitation have been used to ensure oxygenation, allowing longer culture periods (Qian et al., 2016). To induce the vascularization of brain organoids, Pham *et al.* embedded endothelial cells from patients on 34-day older iPSC organoids with Matrigel^®^. These organoids showed robust vascularization ether during the 3–5 weeks of *in vitro* culture and 2 weeks after transplantation on mice (Pham et al., 2018). Another disadvantage of organoids is the lack of control of the shape and size when forming the structures since the organoid replicas must be as similar to each other as possible to validate the results observed from these experiments (Jeong et al., 2022).

Another complexity of the use of these cell-based models is the difficulty of real-time optical monitoring. Live or time-lapsing imaging is possible but to assess the center of the aggregate, researchers use histological and immunohistochemical analysis (Lancaster et al., 2017), meaning that the organoid has to be cut into slices to allow better diffusion of the staining agents and microscope visualization (Lancaster and Knoblich, 2014).

#### Use of 3D (Bio) printing to build 3D models

Among the fabrication techniques for developing 3D models, 3D printing arises as an innovative and useful tool to produce scaffolds. It is described as the process where thin layers are deposited in a substrate to be cured later, producing a 3D structure. When referring to bioprinting, cells and materials are printed together. One of the most interesting features of this bioprinting approach for neural models is the precise control of the deposition site of both materials and cells, allowing the recreation of the brain tissue architecture more realistically (de la Vega et al., 2019).

Several critical parameters must be taken into consideration when engaging in bioprinting for neural models, including the selection of ink with its relevant rheological properties, nozzle diameter, operational temperature, and radiation. (Ding et al., 2018; Heidarian et al., 2019). The printing process may lead to reduced cell viability, particularly when working with sensitive cell types like neural cells, especially when rigid materials are employed. To mitigate this, the use of shear-thinning bioinks, such as hydrogels, is preferable, as they offer better cell protection. Neural cells exhibit a preference for softer biomaterials as substrates or scaffolds, and hydrogels present favorable mechanical properties and a wide range of available polymers, making them suitable candidates (Cadena et al., 2021).

**FIGURE 2:**
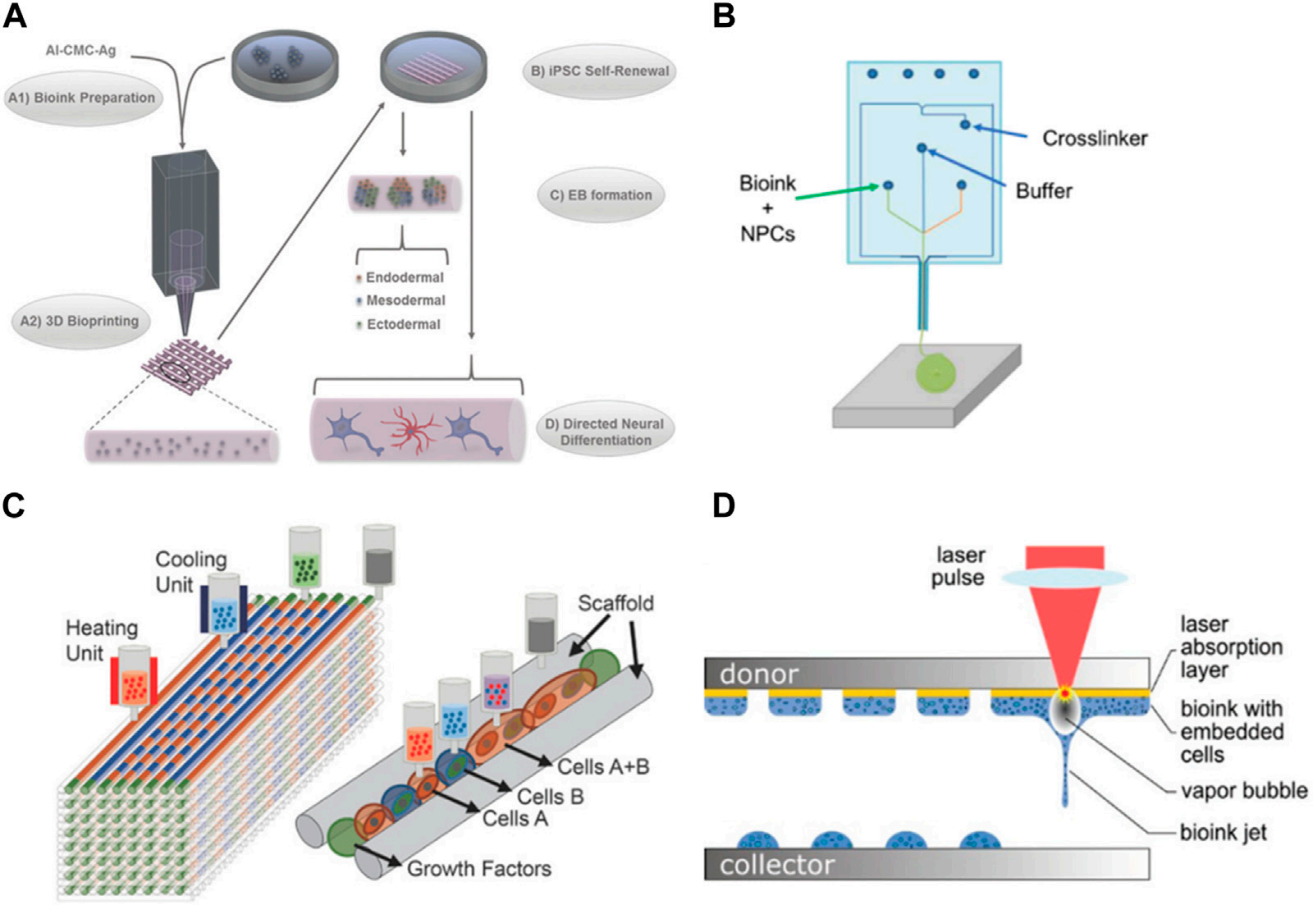
Examples of bioprinting for neural models. **(A)** Bioprinting of iPSC cells in agglomerated to form embryonic bodies and later differentiated in several neural cell types (Gu et al., 2017). **(B)** Microfluidic extrusion used to quickly print iPSC-derived neuronal progenitors (de la Vega et al., 2018) **(C)** Different cell types and hydrogels used to replicate a spinal cord injury (Joung et al., 2018); **(D)** Laser bioprinting of iPSC-derived NSC and neurons (Koch et al., 2023b). Images were reprinted with permission from references.

In addition to the choice of material, these bioinks can be enriched with bioactive cues to enhance neural cell survival, proliferation, and differentiation. Utilizing materials with a low Young’s modulus may compromise scaffold integrity, impeding the creation of layered structures or achieving substantial dimensions in 3D models (Her et al., 2013). The recurrent issue of structural collapse presents a challenge, particularly when working with soft materials, thereby complicating the construction of scaffolds. (Hospodiuk et al., 2017; Oliveira et al., 2019). The nozzle diameter is also important since small diameters allow a better resolution but might suffer clogging (Chang et al., 2008; Malda et al., 2013; Xu et al., 2019). Temperature and radiation also must be adequate to not harm the cells during the process. Particularly print with temperatures compatible with cell culture (close to 37°C) and a short exposure time to radiation. (Nahmias and Odde, 2006; Schiele et al., 2010). Of the several techniques that can be used in bioprinting, extrusion-based bioprinting is the most used technique (Ma et al., 2018). It is the one that applies less pressure, making it safer for the cells and avoiding the dispersion of them into the edge of the construct which can lead to a non-homogeneous distribution of cells (Kabirian and Mozafari, 2019). Gu et al. successfully bioprinted human iPSCs with a polysaccharide-based bioink using an extrusion method. This allowed the proliferation and differentiation of the iPSCs into neuronal subtypes and supporting microglia (Figure 2A). This bioprinted scaffold proved to be a useful model for performing drug screening in addition to the disease models (Gu et al., 2017). For neurodegenerative disease modeling, Zhang *et al.* used extrusion bioprinting to develop a 3D core-shell model to simulate AD neural tissue. They utilized NSC with FAD mutation embedded in Matrigel as the core bioink and alginate as the shell bioink. These structures demonstrated tendencies to self-cluster, maintaining high viability over a long-term period while displaying enhanced differentiation in comparison to 2D models. The engineered construct significantly affected the NSC growth, leading to heightened aggregation of Aβ as well as its expression together with tau isoform genes, when compared to 2D models (Zhang et al., 2022).

The microfluidic extrusion is a similar technique where the bioink and the crosslinking agent meet in a microfluidic chamber before extrusion, polymerizing before the deposition which allows an easy flow when passing through the nozzle, printing more defined structures (Thomas and Willerth, 2017). De la Vega et al. used extrusion to print hiPSC-derived neural progenitor cells in under 5 min while maintaining the viability and differentiation capacity of the cells to generate spinal cord motor neurons (Figure 2B). This bioprinter can process the materials before printing, allowing the programming of the cell-laden bioinks patterns which is an advantageous tool to control the deposition of the cells in an architecture close to the brain tissue. Cells were cultured for a month and exposed to small molecules (SB431542 (SB), CHIR99021 (CHIR), LDN-193189 (LDN), retinoic acid (RA), and purmorphamine (Puro)) to evaluate their effect during the differentiation process (de la Vega et al., 2018).

One of the advantages of using bioprinting for the development of neural models is that it allows printing multiple cell types as you can load different bioinks in independent print heads. (Knowlton et al., 2018). Culturing multiple cell types is of great interest in brain models. Printing neurons together with glia cells (astrocytes and pericytes) would produce a more complex model where the interaction between the different cells can be studied, including the influence of glia cells in the development and survival of neurons. Joung *et al.* were able to create a 3D bioprinted platform that incorporates iPSC-derived spinal neuronal progenitor cells and oligodendrocytes progenitor cells capable of a later differentiation to spinal neurons and oligodendrocytes (Figure 2C). iPSC-derived spinal neural progenitor cells and oligodendrocytes progenitor cells clusters were bioprinted through extrusion-based multimaterial 3D bioprinting, allowing precise control over the position of these cell agglomerates and the use of different bioinks in different print heads. These oligodendrocytes were capable of myelinating the axons and providing a model for damaged central nervous system tissue (Joung et al., 2018). Inkjet bio printing is another technique that can be used to print neural cultures, including multiple cell types. It uses forces such as thermal, acoustic, piezoelectric, or electro to eject the drops of the bioink into the substrate (Gudapati et al., 2016). Its main advantages are the lower cost when compared with other techniques, high printing speeds, and high spatial resolution. However, it is limited to printing low-viscosity bioinks and low cell density as the clogging of the nozzle is common, being suitable for low thermal bioinks as well (Heidarian et al., 2019). Sullivan *et al.* used a commercial bioprinter to deposit nanoliter droplets of a PEG-based matrix, supplemented with RGD and Tyr-Ile-Gly-Ser-Arg peptide motifs and collagen IV to create a 3D model using iPSC-derived cells for neural culture. IPSC-derived astrocytes, neural progenitors. Brain endothelial-like cells and neurons were found viable when cultured in this matrix and the system allowed the endothelial-like vasculogenesis and improved neural differentiation and spontaneous activity (Sullivan et al., 2023).

Other works used laser-based bioprinting for neural cell culture. This technique is based on a laser beam focused on an absorbing layer that propels the bioink toward the collector. It maintains a high resolution with a medium/high speed of printing; however, it is expensive when compared to other bioprinting techniques. In addition, both, the laser and the dropping force, can cause cell death (Sarker et al., 2019). Koch *et al.* used laser-based bioprinting to compare parameters such as viability, differentiation potential, and functionality of both iPSC-derived neural stem cells and neural differentiated NSCs (Figure 2D). Results showed a higher viability after printing of NSC in comparison with printed differentiated neurons. The frequency and intensity of the activity were also superior in NSC culture. The co-culture with astrocytes was also evaluated and proven to further support the network formation and collective activity in NSC culture (Koch et al., 2023a).

Another great advantage of printing different cell types is the possibility of printing endothelial cells in the same construct as neural cells (Sullivan et al., 2023). The vasculature is essential for tissue survival and the including of it in an *in vitro* model contributes to the addition of more physiological relevant features while improving the culture of neural cells. Another important advantage is the improve of the culture time of these 3D constructs. Since large bioprinted structures can have their diffusion of media limited, the creation of vasculature within the model would allow an extension of the time of culture. This extension of culture protocols is advantageous not only for the completion of the long-lasting differentiation protocols of iPSC but also to better mimic late-set diseases such as neurodegenerative diseases (Cadena et al., 2021). Skylar-Scott *et al.* bioprinted iPSC cells to create a layered structure with NSC, endothelium, and neurons, resulting in vascularized and patterned cortical organoids within days (Skylar-Scott et al., 2022).

#### Use of microfluidic devices for the development of brain-on-a-chip models: organ-on-a-chip approach

The brain models discussed above are stationary and have certain constraints. To introduce dynamic features to neural models, among other characteristics, microfluidic platforms are an interesting alternative. By using these small sized platforms, it is possible to create models with a tunable framework and fluid flow withing micro-compartmentalized devices (Bhatia and Ingber, 2014). Culturing different types of cells together in other types of *in vitro* models can be a challenge because one type of media cannot be suitable for all cells. However, microfluidic devices can assist in this matter as their structure allows the introduction of different fluids, minimizing the mixing effect, and allowing each cell type to receive its corresponding medium (Achyuta et al., 2013). Thus, enabling the culture of different cell types as they are seeded in different compartments that are interconnected and allowing the study of their interaction without risking cell viability. Another advantage of this type of cell culture is the compatibility with inspection techniques such as optical or high-resolution video microscopy allowing easier monitoring of the culture over time due to the transparency of the materials chosen for the fabrication of this microfluidic device (Wang Z. et al., 2018; Moutaux et al., 2018).

Cells can be easily cultured inside the device and form a simple monolayer on the base of a microchannel (Taylor et al., 2005). These 2D microfluidic devices still present an increased complexity of the system, compared to a regular 2D culture dish, because or their architecture and ability to include co-culture, fluid flow, and cell response to shear stress. Other times cells are embedded in hydrogel to recreate a more 3D dimensional model, where the advantages of organ-on-a-chip and 3D models can be combined (Koo et al., 2018a).

The ability to modulate the architecture of the channels in a chip allows some control of the cell evolution, such as in the direction of growth or migration (Oddo et al., 2019). Within the various channel distributions that this type of device can take, the most popular used architectures are stack and parallel formation (Amirifar et al., 2022). The stack is when two channels are vertically aligned and separated by a porous membrane. It is a very common architecture for replicating the blood-brain barrier (BBB) and studying drug permeation, cell migration, and/or invasion, among others. (Kilic et al., 2016; Motallebnejad et al., 2019a; Staicu et al., 2021; Fanizza et al., 2022). The parallel structure is when the channels are aligned parallelly to each other and separated by small pillars. This architecture is often used with hydrogels and cells embedded in the central microchannel (Adriani et al., 2017; Osaki et al., 2018a; Campisi et al., 2018).

**FIGURE 3:**
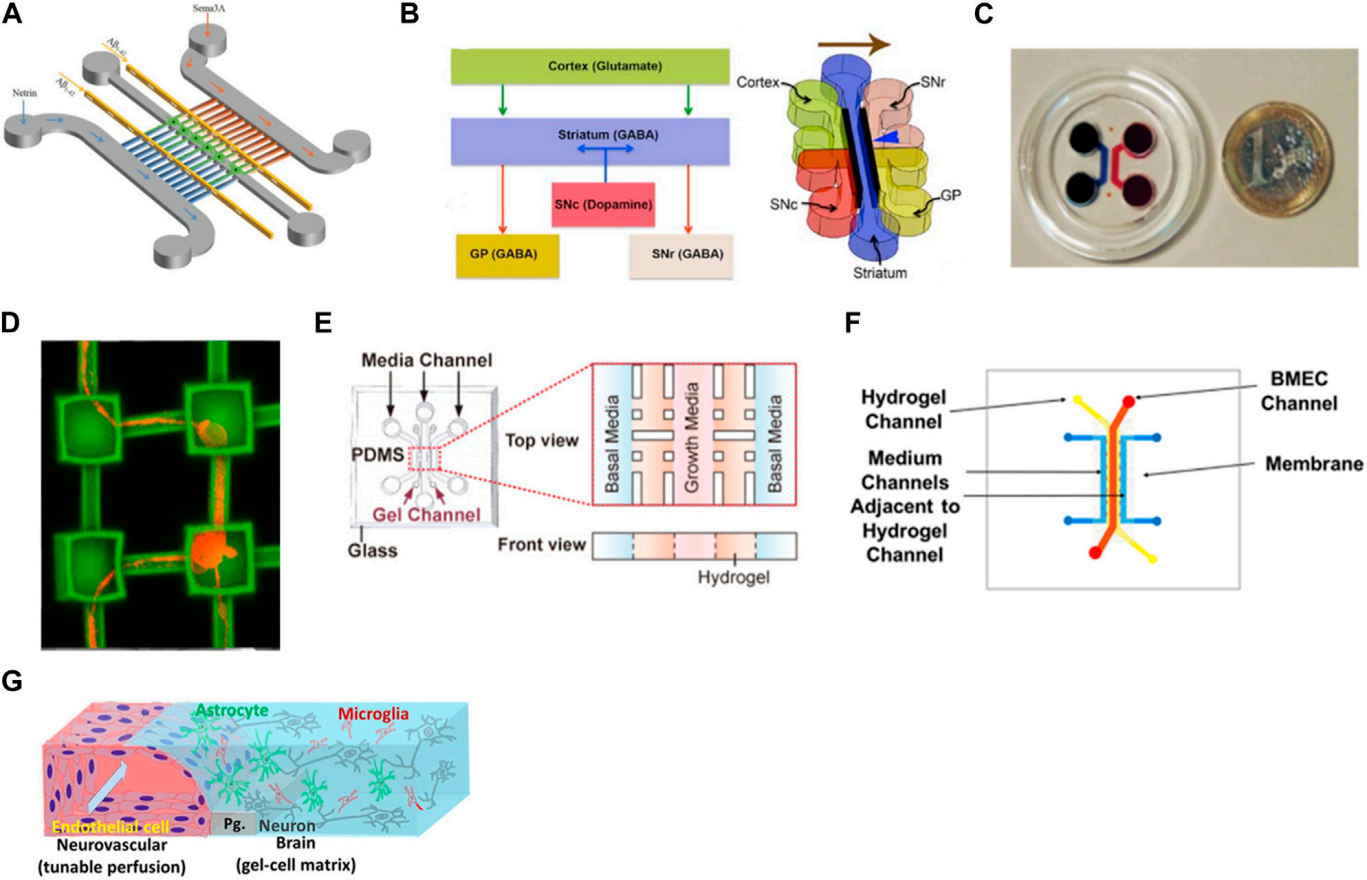
Examples of microfluidic device designs for brain modeling. **(A)** A device with two cell chambers divided with microchannels with a synaptic chamber in between for Alzheimer’s disease (Li et al., 2017). **(B)** Multichambered device for culture of different types of neurons connected through microchannels (Roach et al., 2019). **(C)** Microfluidic-assisted axotomy device to study the regenerative ability of motor neurons after lesion (Sala-Jarque et al., 2020); **(D)** Brain-on-a-chip composed of microcompartments where hiPSC-derived neurons were cultured and formed networks (Harberts et al., 2020). **(E)** 3D BBB model with human endothelial cells embedded in Matrigel^®^(Chung et al., 2020); **(F)** Sandwich-type microfluidic device with an endothelial layer of cells cultured on the top channel and separated with a porous membrane from a bottom layer where astrocytes were seeded in a 3D matrix (Motallebnejad et al., 2019b). **(G)** BBB-model where endothelial cells were seeded in close contact with neurons, astrocytes, and microglia, creating a 3D tetra-culture brain model (Koo et al., 2018c). Images were reprinted with permission from references.

In neuronal culture, it is common the presence of narrow microchannels between two cell culture chambers. These microchannels are usually designed with 5–10 µm of width to allow only the axons to pass since the cell body usually has bigger dimensions (around 20 µm), enabling the study of the axonal growth, signal propagation, and cell-cell interaction (Taylor et al., 2005; Coquinco et al., 2014). These types of devices can be used for the study of only 1 cell type and the axon’s behavior when exposed to certain substances or they can be used to study the interaction of two or more cell types by seeding the cells in interconnected chambers (Roach et al., 2019). Virlogeux *et al.* used a three-compartment device connected by microchannels to recapitulate both the healthy and Huntington’s disease (HD) corticostriatal network. They use the chambers and channels to create a presynaptic, synaptic, and postsynaptic region. Results showed dysfunctions and hypersynchrony on HD devices (Virlogeux et al., 2018). Li et al. also used a three-compartment chip but this time to study the neurotoxicity of Aβ peptides, very characteristic of Alzheimer’s disease (Figure 3A). The authors integrated two microgrooves connected to the compartments to create a gradient of chemotactic factors. These devices allowed the demonstration of a localized mechanism for the neurotoxicity induced by Aβ peptides, useful for the development of Alzheimer’s disease treatments (Li et al., 2017). Roach *et al.* developed a microfluidic device to mimic the complex brain circuitry with 5 chambers where different sub-types of neurons were culture, and connected by microchannels (Figure 3B). The chip proved to recapitulate the formation of an active neural network with normal cell morphology (Roach et al., 2019). Sala-Jarque *et al.* developed a microfluidic device composed of two chambers separated by microchannels to mimic the neuromuscular junction on peripheral nerve injuries (Figure 3C). Motor neurons were cultured on one side while muscle cells were on the other. A perpendicular channel between chambers and passing through all microchannels was used to vacuum-induce axotomy through air bubbles. The regenerative abilities of the neurons were assessed after optogenetic stimulation both on neurons and muscle cells. Results showed an increased axon regeneration after increasing neuronal activity as well as a release of paracrine factors by the muscle cells after stimulation, also triggering the regrowth of axons on the lesion area (Sala-Jarque et al., 2020). Another model of the neuromuscular junction used a similar microfluidic platform to differentiate healthy and amyotrophic lateral sclerosis hNSC into proprioceptive sensory neurons, co-cultured with motor neurons. Results demonstrated that the cells were able to interact by the formation of annulospiral wrapping-like structures. The genetic profile comparison between healthy and ALS samples pointed to lower values of ETV1 (important for motor feedback) which highlights the involvement of proprioceptive sensory neurons in this pathology (Badiola-Mateos et al., 2022). Haberts *et al.* also developed a brain-on-a-chip where microchannels connected different compartments (Figure 3D). In this design, elevated cavities were produced through 3D nanoprinting and interconnected by microchannels to form a complex structure. Human iPSC-derived neurons were cultured in this model and the neural outgrowth, network size, and branching behavior were evaluated. Results showed a developed neural network with functional activity after only a few days in culture, validating the device design (Harberts et al., 2020).

The separation between the axons and the cell body not only can be done using the microchannels mentioned above but also through pillars with embedded hydrogel (Adriani et al., 2017; Wang Y. et al., 2018; Kamande et al., 2019; Klim et al., 2019). Chung *et al.* used 3 channels where brain endothelial cells were cultured on the middle one for blood-brain barrier modeling (Figure 3E). The receptor-mediated transcytosis, as well as brain endothelial-specific penetrating abilities of different peptides, were evaluated and the models were proven to be used for drug development (Chung et al., 2020). Osaki *et al.* used a compartmentalized model to study amyotrophic lateral sclerosis by culturing iPSC-derived motor neurons from patients in the form of spheroids together with skeletal muscle bundles. The results showed the neuromuscular junctions between the axons and the muscle fibers. It also demonstrated fewer muscle contractions, motor neuron degradation, and increased apoptosis of the muscle on the ALS-motor unit, compared to non-ALS devices (Osaki et al., 2018b).

Static microfluidic platform culture models present interesting features for modeling healthy and diseased tissue since their architecture is still innovative. An example is the model developed by Park et al. that incorporates three different cell types in a 3D architecture for Alzheimer’s disease modeling in a no-flow experiment (Park et al., 2018). Neurons, astrocytes, and microglia cells derived from iPSC were chosen to recapitulate representative features of Alzheimer’s disease such as beta-amyloid aggregation, neuroinflammatory activity, and phosphorylated tau accumulation. However, the inclusion of perfusion in a cell culture model is, as mentioned above, a great advantage of these platforms. The presence of fluid flow improves the diffusion of media through the culture as well as exposes the cells to shear stress which influences cell morphology, behavior, and gene and protein expression (Huang et al., 2022).

Uzel *et al.* designed two microfluidic devices capable of generating orthogonal gradients within a gel region and studied the ability of two different molecules to induce the cellular response of mouse embryonic stem cells (Uzel et al., 2016). The presence of a gradient of molecule concentrations in a cell culture model is important because it influences cell behaviors such as differentiation and maturation (Kilic et al., 2016). In static conditions, the design of the device allows the insertion of gradients that can influence the proliferation and differentiation of cells (Park et al., 2009). However, by taking advantage of the perfusion that can be used in a microfluidic device, the exposure of cells to different gradient concentrations can be easily performed (Demers et al., 2016; Uzel et al., 2016).

Looking at the bibliography, we can find a superior number of publications on static devices than on dynamic ones. This is mainly due to the difficulty of introducing fluid flow in neuronal culture and the physiology of the brain tissue itself. Cells in the brain are extremely sensitive, especially primary cultures or induced stem cells. A harsh small movement in the seeding or maintenance of the culture can lead to cell detachment and subsequent loss of the cells in the culture. Inclusive, high-shear stress can be used to model traumatic brain injury, for example, by including a channel that crosses the microchannels where the axons are located where a high fluid flow is applied, damaging the cultured axons and thus simulating the injury (Cullen and LaPlaca, 2006)). A possible solution to minimize this effect is to combine the organoid culture with the microfluidic one. iPSC in organoids presents higher resistance to shear stress and fluid flow can be easily incorporated at the embryonic body stage of differentiation, which would also be advantageous to the perfusion problem that organoid culture faces (Park et al., 2015; Wang Y. et al., 2018).

Regarding the tissue, neuronal cells are fed by the brain vasculature. This vasculature is different from the other vasculature in the body due to the BBB present in every blood vessel in the brain. This tight unit is built not only by endothelial but also by neural cells such as astrocytes and pericytes (Chow and Gu, 2015). When planning the design of a microfluidic model, the most physiological way to mimic the fluid that nourishes the neurons is to mimic the BBB. So, there are several models of BBB-on-a-chip that besides culturing the different cell types present in the NVU, also introduce fluid flow in the channels with endothelial cell culture (Achyuta et al., 2013; Deosarkar et al., 2015; Adriani et al., 2017; Bang et al., 2017; Sances et al., 2018a; Campisi et al., 2018; Vatine et al., 2019a; Chung et al., 2020) Sances et al. developed a spinal cord-chip system with iPSC-derived spinal neural progenitor cells cultured with brain microvascular endothelial cells (Sances et al., 2018b). This co-culture model had increased neuronal activity in vascular-neural interaction genes, showing a developmental gene expression closer to what happens *in vivo*. Vatine et al. used a BBB-on-a-chip with co-culture of neural and endothelial cells, and perfusion which enhanced the performance of the barrier, exhibiting physiologically relevant TEER values (Vatine et al., 2019b). The model was able to replicate the inflammatory response and transport of biomarkers. When using cells from patients, the model could detect functional differences when compared to healthy donors. Motallebnejad *et al.* developed a BBB model where hiPSC-derived brain microvascular endothelial cells were cultured in a top channel, connected through a porous membrane to a lower channel where astrocytes embedded in a hydrogel were seeded (Figure 3F). The presence of tight junction proteins and permeability studies confirmed the integrity of the BBB. Moreover, by adding TGF-β1 the integrity of the BBB was disrupted, contributing to the versatility of this model (Motallebnejad et al., 2019a). Koo *et al.* created a 3D BBB-on-a-chip using the commercially available device from Mimetas (OrganoPlate) with four different cell types (Figure 3G). Endothelial cells were seeded in direct contact with neuroblastoma, microglia, and astrocytes embedded in a hydrogel. This model was used to assess the neurotoxicity of organophosphate-based compounds (OPs) and results demonstrated the permeation of OPs through the BBB and inhibited the acetylcholinesterase activity. These results correlated with *in vivo* data, validating the potential of this device (Koo et al., 2018b).

Despite the many advantages of microfluidic devices, they still present some limitations. The handling of these devices is not easy as they can face throughput and reproducibility issues. In addition, it is difficult to have control over the properties and microstructure of the devices in every manual replica. Clogging of the channels with cells, debris, or bubbles influences their functioning and the shear stress must be applied carefully to not harm the cells in culture (Bhatia and Ingber, 2014). Even though, parallel formations with transparent materials such as PDMS facilitate the visualization of the cells in culture and the incorporation of sensors in the design of the device facilitates the analysis during culture time, (Kuswandi et al., 2007).

Recent models indicate that the future of 3D culture will be related to the integration of microfluidic devices, hydrogels, and/or spheroids/organoids to evolve these devices into better mimicking models (Liu et al., 2019). As mentioned before, organoids are widely used as 3D brain models to study neurodevelopment or brain diseases. One of the biggest disadvantages of this culture is the necrotic core that happens when the size of the organoid increases and the diffusion of nutrients and oxygen becomes difficult. The combination of microfluidic devices in organoid culture is a possible solution to allow the continuous exchange of cell culture medium without the need for big reactors. Furthermore, the ability to control the organoid culture with microfluidic devices allows a less heterogeneous production of these agglomerates, another common downside. Wang *et al.* used a microfluidic platform for brain organoid culture to evaluate the effects of nicotine exposure on prenatal brain development. The device was composed of five channels, two with the iPSC-derived organoids embedded in Matrigel^®^ and the other three for fluid flow (Wang Y. et al., 2018). The dimensionality of the organoids and fluid flow increased the cell viability and marker expression in comparison with the 2D culture.

Taking advantage of the possibility of compartmentalization and fluid flow, these platforms incorporate one or several channels surrounded by fluid channels to allow the exchange of nutrients and oxygen. Adriani *et al.* built a 3D neurovascular microfluidic model with 4 channels separated by pillars to mimic the neurovascular unit (Adriani et al., 2017). The channels included medium, primary rat astrocytes embedded in the hydrogel, primary rat neurons embedded in the hydrogel, and human endothelial cells plus medium. The cells presented type-specific morphology and functional properties similar to other existing models. Lee *et al.* developed a microfluidic device of 3 channels separated with pillars where hiPSC-derived endothelial cells, primary brain pericytes, and astrocytes were cultured with a fibrin hydrogel to quantify nanoparticle permeability. Surface-functionalized particles showed a higher permeability than non-functionalized ones. This model enabled rapid analysis of the permeability compared to transwells models and with a more physiological relevance (Lee et al., 2020).

Novak *et al.* developed a robotic platform that links 8 different types of organs-on-chips, using what they called the Interrogator (Novak et al., 2019), to create a complex model. This completely automatic platform allows continuous liquid transfer from one organ-on-a-chip to the other with *in situ* imaging. Relating this type of model with a disease can be of extreme value in evaluating not only the side effects of a drug for its treatment but also the influence of a diseased organ on the other healthy organs.

Characterization of 2D models by immunochemical staining, electrophysical measurements or biochemical analysis is well described but characterizing 3D microenvironments is still challenging. There is a limitation for the detection and monitorization of 3D features such as the synaptic activity of brain models. The possibility of using microfluidic devices with electrophysiological sensors is another promising integration when performing neuronal culture (Habibey et al., 2017). Honrado *et al.* combined neuronal networks with a microfluidic device for impedance cytometry capable of predicting cell size, cross-sectional position, and velocity (Honrado et al., 2020). This is an example of how microfluidic devices can be used in high-throughput measurements useful for drug testing, diagnosis, and personalized medicine. Grist *et al.* created a microfluidic platform that combined spatiotemporal oxygen control and long-term microscopy to assess tumor spheroid response to hypoxia (Grist et al., 2023). Results showed the accumulation of doxorubicin in tumor spheroids is cycling hypoxia dependent as well as the size of the tumor during treatment due to the oxygen control the microfluidic platform allowed as well as the long-term two-photon microscopy monitoring. The conjugation of electrodes is very useful to monitor if the network is functional and acting according to healthy or diseased circumstances. Palma-Florez *et al.* used a microfluidic platform with a trans-endothelial electrical resistance measurement system to evaluate the permeability of a therapeutic nanosystem for AD treatment. Human astrocytes and pericytes were cultured in the central channel while endothelial cells were placed in the lateral channel. The nanoparticles were able to permeate through the barrier, particularly the ones containing angiopep-2 peptide, a molecule that has been shown to help in the disaggregation of the amyloid. This platform was functional for the study of nanotherapeutics’ effects on brain permeability (Palma-Florez et al., 2023).

### Integration of 3D (bio)printing with 3D cell cultures and microfluidic platforms

A feature of 3D printing available to incorporate into microfluidic devices is bioprinting. A way to do this is using different print heads, one for the material of the device and the other(s) for bioink(s) with different types of cells. The structural material of the device can be cured in advance, so cells are not harmed during the curing process. A more accurate and controlled positioning of the cells within the model would be beneficial, especially when taking into consideration the different placement of neural cells within the brain. Considering the brain has zones with different percentages of various cell types and that most models do not use multi-cell type culture and the spatial distribution is aleatory, bioprinting arises as a promising tool to help build an accurate brain model, with co-culture but also with the disposition of cells corresponding to the different brain regions. Johnson *et al.* used an approach based on addictive manufacture to produce a 3D printed nervous system on a chip. Through micro-extrusion, microchannels and chambers were printed, mimicking the communication between the glial cells and axons in the nervous system to test nervous system infections. This platform was a successful multiscale and biomimetic model (Johnson et al., 2016). Yi *et al.* created a microfluidic device to study glioblastoma using bioprinting which resulted in a model composed of patient-derived glioblastoma, vascular endothelial cells and decellularized ECM from porcine brain capable of recreating features of this tumor to be used as drug testing platform (Yi et al., 2019). Kajtez *et al.* developed a 3D printing soft-lithography technique to print a device for neuronal culture. The printed platform stands for the long-term culture of human stem cell-derived neurons and astrocytes. The authors also used this platform to modulate the nigrostriatal pathway in Parkinson’s disease and were proven successful in maximizing the unidirectional growth of the dopaminergic projections (Kajtez et al., 2020).

We can find some examples of organoids’ growth inside microfluidic devices to improve their perfusion and vascularization procedures. The incorporation of better characterization techniques or sensors is also being studied and integrated into recent models. Combining bioprinting with the microfabrication process of organs-on-chips can be a way to improve the mechanical properties as well as the porosity, microstructure, and even the polymerization mechanisms of the material chosen, for example, a hydrogel. This combination allows control over the composition of the device to better mimic the tissue. It is also possible to introduce growth factors, cytokines, or other molecules in the bioink and place them directly where it is desired. Printing cells into these devices would decrease the time and complexity of cell seeding in a chip. The possibility of creating a vascular network is another attractive option (Yu and Choudhury, 2019)**.**

## Conclusion and future perspectives

Neurodegenerative diseases affect a high percentage of elderly people around the world and due to the increasing in the age of the population, the number is estimated to advance rapidly. When animal models and traditional 2D culture are not enough to answer how to treat these conditions, more elaborate models are needed, recognizing the importance of tissue architecture and the use of more specific cells. It is critical to correctly mimic the communication that undergoes between all the different cell types present in the brain, not only in the case of a healthy situation but also in the case of a disease. To do so, induced pluripotent stem cells arise as an accurate choice since they are obtained from the patient and can create all the cell types from the three germ layers. This type of cell is then considered the future of *in vitro* modeling and their combination with 3D models is on the edge of the research.

However, neurodegenerative cell culture models still face some challenges. The time of culture is one of them when considering iPSC. The model must guarantee cell survival/viability and function for as long as the experiment is intended. Most times the model looks very promising but then the cells are only alive for a few hours or days which does not allow the mimicking of long-term diseases or their effects (Kelava and Lancaster, 2016b). Other challenges in 3D are the compatibility with live imaging techniques or the difficulty in the perfusion of nutrients and oxygen. Recently has been proposed 4D bioprinting as a cutting-edge additive manufacturing technology that has an intrinsic capability to fabricate *de novo* living tissue constructs which can be made to change in various mechanical or physio-spatial aspects when subjected to predetermined stimuli or trigger sources. 4D bioprinting techniques can be used to place both living cells and growth factors in highly ordered, biomimetic motifs which can undergo physiologically relevant transformations to accurately simulate developmental processes, such as tissue stretching, compression, or shifting of the biomaterial’s modulus. The fourth dimension in “4D bioprinting” refers to the time element on which one or more of a 3D printed object’s physical attributes are functionally dependent (Esworthy et al., 2019). New techniques developed recently show the feasibility of manufacturing cerebral organoids and assembloids by mimicking human organogenesis. However, the bottom-up technology to produce organoids still requires further work to advance functional maturation and assemble neural circuits. Multilineage assembloids could provide a more complex system including blood vessels although the process is very difficult to standardize. In this sense, some future improvements would include: i) the development of techniques to bioprint hNPCs that will go for terminal differentiation in the 3D structure. ii) Demonstration of the selective effect of bio-inks with natural ECM on neuronal differentiation. iii) Development of the techniques to bioprint blood vessels with artificial- or natural-derived bio-inks and iv) Improve 4D bioprinting techniques to bioprint all components of the brain organoids.

The perfect *in vitro* model for neurodegenerative disease needs still to be found but combined efforts of all these years have shown that we are going in the right direction and that there is still hope to find a more accurate model to better understand these diseases, how and when they appear, how they evolve and maybe, help in finding a better drug to treat them. Learning from every downside of the existing models is crucial to tune the right direction. The perfect scenario is one where we can combine all their advantages to include the self-organization of the cells occurring in organoids with the precise control over cell seeding and scaffolds that bioprinting allows or the compartmentalization combined with the possibility of recreating mechanical forces that microfluidic devices possess.
